# Endometrial Glandular Dysplasia (EmGD): morphologically and biologically distinctive putative precursor lesions of Type II endometrial cancers

**DOI:** 10.1186/1746-1596-3-6

**Published:** 2008-02-08

**Authors:** Oluwole Fadare, Wenxin Zheng

**Affiliations:** 1 Department of Pathology, Wilford Hall Medical Center, Lackland Air Force Base, San Antonio, Texas, USA; 2 Department of Pathology, University of Texas Health Science Center at San Antonio, San Antonio, Texas, USA; 3 Department of Pathology, Department of Obstetrics and Gynecology, and the Arizona Cancer Center, University of Arizona College of Medicine, Tucson, Arizona, USA; 4 Department of Pathology and Hospital of Obstetrics and Gynecology, Shanghai Medical College, Fudan University, Shanghai, China

## Abstract

In this article, the authors briefly review the historical evolution of the various putative precursor lesions for Type II endometrial cancers, with an emphasis on the newly defined "Endometrial Glandular Dysplasia (EmGD)". The evidentiary basis for delineating serous EmGD as the most probable precursor lesions to endometrial serous carcinoma is reviewed in detail. An argument is advanced for the discontinuation of the term serous "endometrial intraepithelial carcinoma (EIC)" as a descriptor for a supposedly intraepithelial, precancerous lesion. Preliminary evidence is also presented that suggests that there is a morphologically recognizable "clear cell EmGD" that probably represents a precancerous lesion to endometrial clear cell carcinomas.

## Background

Endometrial cancers are the most frequently diagnosed malignancies of the female genital tract in the United States, with 39,080 new cases projected for 2007 [1]. Since 1983, two broad clinicopathologic subtypes of endometrial carcinomas have been recognized [2]. This conceptual classification has largely been supported by subsequent molecular-cytogenetic data, which has facilitated the acceptance of the so-called *dualistic *model of endometrial carcinogenesis [3-8]. Type I cancers, the prototype of which is the endometrioid histotype, occur in comparatively younger age group [3-8], appear to be related to unopposed estrogen stimulation [9-14], frequently express the estrogen and progesterone receptors [7,13,14], arise in a background of glandular hyperplasia [5,7,13,14], and has a relatively favorable prognostic profile [15]. Genetic alterations in Type 1 cancers include PTEN inactivation [16-19], beta-catenin (CTNNB1) mutations [17], and less frequently, microsatellite instability (related to inactivation of the MLH1 gene) [20,21], and activational mutations of the K-ras gene [22]. Type II cancers, the prototype of which is the endometrial serous carcinoma (ESC), and which was previously termed *uterine papillary serous carcinoma *(UPSC), typically occur in an older age group [3-8], frequently arise in a background of inactive or resting endometrium [3-8], and display a low frequency of expression of hormonal receptors [13,14,23,24]. Type II cancers also display frequent mutation and overexpression of the p53 [24-26] and HER2/neu [27,28] genes and proteins respectively, and have a comparatively poor prognosis independent of other factors [29-32]. This model has provided a valuable framework for the study of various aspects of endometrial carcinogenesis and for the potential development of therapeutic modalities that are pathway specific. Nonetheless, approximately 7400 deaths attributable to uterine corpus malignancies are projected for 2007 [1]. This relatively high mortality rate suggests that prevention and/or early detection remain highly essential approaches to the prevention of endometrial cancer-related mortality. One aspect of cancer prevention is the recognition of morphologically distinctive precursor lesions or "precancers" [33], so that a therapeutic intervention can be administered prior to the development of the well-developed malignancy. For more than 100 years, scientists have noted a spectrum of epithelial changes that have tentatively been considered to be precancerous in nature based on one or more of the following factors: a) the frequent coexistence of the putative precursor lesions with the well-developed malignancy as well as occasional morphologic transitions between them b) Shared epidemiologic, patient demographical, immunophenotypic and/or molecular genetic properties between the putative precursor lesions and their associated well-developed malignancies, and c) Longitudinal follow-up data that suggests that the putative precursor lesions confer an increased risk for the development of invasive malignancies [24,26,32,34-88]. These factors notwithstanding, the definition, full morphologic spectrum (including upper and lower limits) and clinical significance of the various putative endometrial precancers remain controversial. One factor contributing to this state of affairs is the ever-evolving nomenclature of endometrial precancers, a significant impediment to comparing data between studies. The purpose of this commentary is to summarize the current published data that forms the basis for the recent delineation of Endometrial Glandular Dysplasia (EmGD) as the most probable precancerous lesions for serous and probably clear cell carcinomas of the endometrium.

### Serous endometrial glandular dysplasia

Reports describing variably papillary endometrial cancers with psammoma bodies have appeared in the literature since at least 1963 [89-93]. However, it was in the 1980 text by Hendrickson and Kempson that the concept of "serous" differentiation and aggressive behavior in these cancers was first emphasized [56]. Endometrial serous carcinomas (ESC) are now well recognized as uncommon endometrial cancers with distinctive morphologic features and a significantly worse overall survival as compared their endometrioid counterparts [29-32,94]. In 1992, Sherman et al [32] described 32 uterine carcinomas with a serous component, including 13 pure cases and 19 cases admixed with other histotypes. The authors noted the existence of "cytologically malignant cells closely resembling the invasive serous carcinoma in the surface endometrium adjacent to the tumor" [32]. This lesion was present in 89% of their 32 cases and was designated "intraepithelial carcinoma" by the authors [32]. Two reports published in 1995 appeared to be describing essentially the same "intraepithelial" lesion [66,67]. Spiegel et al [67] reviewed 518 hysterectomy specimens with endometrial cancers and found 89 cases "in which there were microscopic foci of malignant epithelium that failed to alter the architecture of an otherwise thin atrophic or weakly proliferative endometrium or endometrial polyp [67]." Sixty-six percent of the cancers associated with these foci had a serous component, and the author applied the designation "endometrial carcinoma *in situ*" [67]. In a study published later that year, Ambros et al [66] introduced the term "endometrial intraepithelial carcinoma" (EIC), and showed that this lesion was frequently and specifically associated with endometrial carcinomas with a serous component. In 1998, Zheng et al [70] used the designation "uterine surface carcinoma" to describe this lesion, noting that it is "often multicentric and behaves in a more aggressive fashion than regular in situ carcinomas" which rendered the previous designations inappropriate. In 2000, Wheeler et al [72] noted the difficulties and questionable validity of distinguishing EIC from stromal invasive but non-myoinvasive (superficial) ESC. The authors proposed the concept of "minimal uterine serous carcinoma", a term that would combine EIC, as previously defined [66], with small superficial ESC (<1 cm). "Serous EIC" is the recommended designation in the most recent WHO classification [88].

Pure serous EIC (serous EIC unassociated with a full blown and/or myoinvasive serous carcinomas) may potentially show extrauterine disease and/or peritoneal carcinomatosis [73]. Shared patterns of p53 mutations between the endometrial and extrauterine lesions argue in support of true origination of the latter from the former [74]. However, since pure serous EIC is so infrequently identified in isolation, there are no systematically collected data on precisely what percentage of them will show extrauterine disease. Non-myoinvasive (stage 1A) ESCs are known to show extrauterine disease in 17–67% of cases [68,76,95,96].

In our opinion, the *serous EIC *designation, as used in this context to describe a supposedly intraepithelial, precancerous lesion, should be discarded. The notion that a "precancerous" lesion may display clinical malignancy (as evidenced by extrauterine extension) is indicative to us of a fundamental fallacy in the definition of the putative precursor lesion and is an obvious contradiction in terms. The distinction between serous EIC (as presently defined) and small "stromal-invasive" (stage 1A) ESC is a rather artificial one that is largely devoid of clinical significance and whose validity is questionable. Both are lined by identical, cytologically malignant cells [32,66,67,70], both may show extrauterine disease [68,76,95,96], and both require comprehensive surgical staging [68,76,81,95,96]. Rather, serous EIC is best conceptualized, and should be clinically managed as, small or early ESC [76]. Clement and Young [97] have outlined a similar approach, writing that they consider EIC "a tiny focus of serous carcinoma and do not qualify it further other than to note its size and location". Although tumor size is a well-established prognostic factor in malignancies of many organ systems, it is unclear if a comparable significance is operational in this particular malignancy, since diffuse extrauterine dissemination has been associated with microscopic endometrial lesions. However, one would intuitively expect the large "malignancy burden" of multifocal or extensive endometrial disease to increase the probability of extrauterine extension. Until the significance of lesion size is clarified, prudence should dictate that lesion size and disease extent be noted in the pathologic report but only *after *properly classifying the lesion as an ESC (rather than a serous EIC).

**Figure 1:**
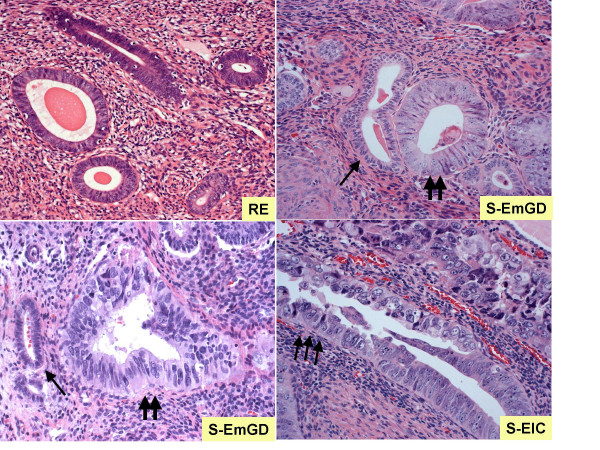
Cytologic features of serous endometrial glandular dysplasia (S-EmGD, 2 arrows), as compared with resting endometrium (RE, 1 arrow) and the so-called serous endometrial intraepithelial carcinoma (S-EIC, 3 arrows).

Given the aforementioned problems with serous EIC as a biologically valid precancer, Zheng et al [79] hypothesized that a morphologically identifiable lesion existed that bridged the gap between resting endometrium, in which ESC most frequently arises, and these so-called serous EIC lesions. The authors examined 108 hysterectomies (32 cases of classic ESC, 16 serous EIC, 60 endometrioid cancers). In 53% of the 32 cases of ESC and 1.7% of the 60 endometrioid cancers (p < 0.0001) one or more microscopic, morphologically atypical lesions that did not qualify for a *serous EIC *designation were identified [79]. These lesions were most commonly single glands or small glandular groups within the superficial endometrium or a flat layer of epithelium on the endometrial surface. These foci did not qualify for a serous EIC designation because their level of atypia was consistently not at the level seen in the adjacent well-developed serous cancers, which is a definitional requirement for serous EIC. These glands were designated "Endometrial Glandular Dysplasia" (EmGD) [79]. The typical EmGD focus showed epithelium lined by cells with nucleomegaly (2–4 times resting endometrium, as compared with 4–5 times in serous EIC), variably conspicuous nucleoli, variable hyperchromasia and loss of nuclear polarity [79] (Figure 1). Notably, the MIB-1 proliferative and p53 staining indices of the EmGD lesions were at least intermediate between the resting endometrium and the serous EIC. Based on their frequent and apparently specific association with ESCs, as well as their "intermediate" morphologic and immunophenotypic features (between benign and malignant epithelium), the authors speculated that EmGD represents the true precursor lesion of ESC [79], on the presumption that endometrial serous carcinogenesis is also a morphologically identifiable "stepwise process", rather than the spontaneous or "*de novo*" arising of ESC from benign endometrium. In a subsequent molecular study, Liang et al [77] provided preliminary evidence that suggested that although EmGD are not lined by comparably cytologically malignant cells, they are closer to EIC/ESC than to resting endometrium. The patterns of loss of heterozygosity at 7 microsatellite polymorphic DNA markers were investigated in laser microdissected areas of EmGD and serous EIC/ESC. For 4 of the 7 markers, the frequency of LOH was higher in EmGD lesions as compared with resting endometrium, reaching statistical significance in 2 (TP53 and D1S162) and approaching such significance in the other 2 (D1S211 and D2S123). By contrast, when EmGD was compared to serous EIC/ESC, in only 1 locus -TP53 – was the frequency of LOH significantly different (31.3% in EmGD versus 60% in serous EIC/ESC). Furthermore, in paired EmGD and serous EIC/ESC samples (from the same patient), there was a high degree of concordance in the frequency of LOH [77]. The frequent mutation of the p53 gene in EmGD was confirmed in a subsequent study [98]. p53 mutations were identified in 0%, 43%, 72%, and 96% of resting endometrium, EmGD, serous EIC and ESC, respectively. Furthermore, in excess of 50% of the uteri with the aforementioned neoplastic lesions showed at least one identical p53 gene mutation among lesions of EmGD, serous EIC and/or ESC [98,99]. These findings provide a molecular link between EmGD and ESC, one of the basic tenets of a putative precancer [33].

Recently, we investigated the endometrial biopsies that preceded hysterectomies in which ESC were diagnosed in an effort to determine whether EmGD lesions could be identified and if so, to estimate the duration between the diagnoses of the dysplastic and malignant lesions [82]. In 250 hysterectomies with ESC, preceding endometrial biopsies were available for evaluation in 27 cases. Of these 27 cases, EmGD lesions were identified in 9 cases (33.3%). The average duration between the biopsies in which the EmGD lesions were identified and the hysterectomies in which the ESCs were diagnosed was 33 months (range 16–98 months). In the control group of 258 hysterectomies with benign diagnoses, only 1 EmGD was identified out of 29 preceding endometrial biopsies that were available for evaluation [82]. This provides preliminary evidence that EmGD may be associated with an increased risk of ESC, another tenet of a precancer [33]. At the protein level, the novel cytoplasmic marker IMP3 [Insulin-like growth factor II (IGF-II) mRNA binding Protein] provides further supportive evidence that links EmGD and ESC. Among endometrial cancers, IMP3 predominantly marks ESC. Zheng et al [100] demonstrated IMP3 expression is present in 14% of EmGD, 89% of serous EIC and 94% of ESC. IMP3 expression was significantly less frequent in the other lesions studied: 0 (0%) of 35, 5 (7%) of 70, 0 (0%) of 8, 3 (25%) of 12, and 5 (33%) of 15 cases of atypical hyperplasia, endometrioid, mucinous, clear cell carcinomas, and other malignancies, respectively.

To summarize the data from our group 1) EmGD is a morphologically identifiable, typically focal lesion that shows an association with endometrial cancers displaying serous differentiation [79]. 2) This association is common (53% of hysterectomies with ESC) and apparently specific, at least relative to endometrioid adenocarcinomas [EmGD was seen in only 1.7% of hysterectomies with endometrioid cancers] [79] 3) EmGD are distinct from ESC/serous EIC as they are lined by cells whose degree of atypia falls short of the frankly malignant cells required of the latter; there are typically no direct morphologic transitions between EmGD and well-developed ESC [76,79]. 4) EmGD lesions display a proliferative and p53 staining index that is at least intermediate between resting endometrium and small ESC/serous EIC, which argues strongly against their being reactive in nature 5) Differential patterns of LOH for selected DNA markers in EmGD, ESC/serous EIC and resting endometrium indicates significant differences between EmGD and the resting endometrium in which they were identified and a kinship with ESC/serous EIC [77]. 6) EmGD was identified in 33% of the endometrial biopsies that preceded hysterectomies with ESC at an average duration of 33 months, but was present in only 3.4% of endometrial biopsies that preceded uteri with benign diagnoses [82]. Combined, these data provide evidence that EmGD is the most likely precursor lesion for ESC. EmGD fulfils most of the National Cancer Institute (NCI)'s criteria for a precancerous lesion [33]: (1) there is preliminary evidence that EmGD is associated with an increased risk of ESC; (2) partial molecular concordance in p53 mutations between EmGD and ESC provides one line of evidence that suggests that the latter arises from the former; (3) EmGD differs from the normal tissue from which it arises, and is morphologically recognizable as such; (4) EmGD is morphologically distinct from ESC, being characterized by cells that although more atypical than the background endometrial cells, cannot be characterized as cytologically malignant. (5) It is diagnosable by morphologic evaluation, with judicious use of immunohistochemical adjuncts such as p53, Ki67 and IMP3 in equivocal cases.

Nevertheless, it is unclear if EmGD represents an obligate precursor, or whether it is reversible. At the present time, however, we strongly advocate the discontinuation of the routine usage of the diagnostic terms "EIC" or "serous EIC". Small localized glandular or surface endometrial foci that are lined by malignant cells of the "serous-type" should simply be referred to as ESC, along with the size and if possible location of the lesion, as others have previously suggested [97].

### Clear cell endometrial glandular dysplasia

**Figure 2:**
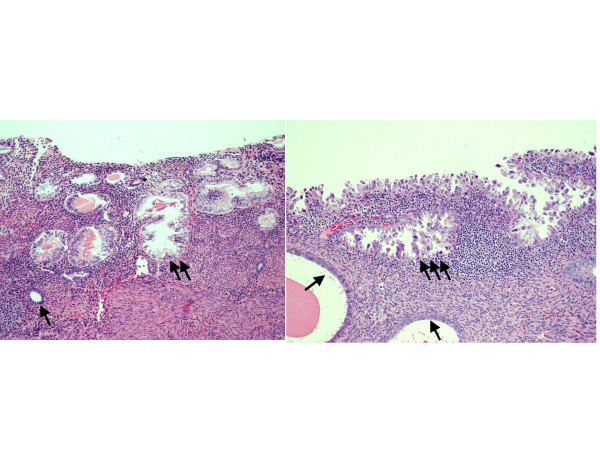
Resting endometrium (1 arrow); clear cell endometrial glandular dysplasia (clear cell-EmGD, 2 arrows); clear cell endometrial intraepithelial carcinoma (clear cell-EIC, 3 arrows).

Endometrial clear cell carcinoma (ECCC), a Type II cancer under the dualistic model, represents 1–5.5% of endometrial cancers [97,101-107]. Given the significant overlap that exists between ECCC and ESC in their morphologic [24,32], clinical [108] and global gene expression [109] attributes, it can be anticipated that an *in situ *lesion probably exists for ECCC. However, in contrast to the cervix, where sporadic examples of clear cell *in situ *lesions have long been reported [110-112], very little had been published on putative precursor lesions for ECCC until recently [86,87]. In 2004, Moid and Berezowski [86] described a distinctive lesion in the hysterectomy specimen of a 70-year-old woman which they designated "EIC, clear cell type". The lesion was comprised of surface epithelium and some glands that were lined by cells with "clear cytoplasm, marked nuclear pleomorphism, coarse chromatin, irregular nuclear membranes, and prominent eosinophilic nucleoli" and which occasionally had a hobnail appearance. The lesions showed "focal" staining for p53, a "moderate to high proliferative index", and no evidence of extrauterine extension [86]. Recently, our group attempted to more systematically characterize the clinicopathologic features of these putative precursor lesions. We evaluated the adjacent benign endometria in 14 cases of pure ECCC and 16 endometrial carcinomas with a greater than 10% ECCC component, in search of lesions that notably stood out from the background benign endometrium [87]. The lesions that we identified were single glands, small glandular clusters or segments of surface endometrium lined by cells that typically displayed cytoplasmic clarity and/or eosinophilia and a continuous gradient of nuclear atypicality. Based on the severity of the nuclear changes, we graded the lesions on a 3-tiered scale, with the grade 3 lesions essentially being lined by frankly malignant cells comparable to those of the adjacent malignancies. Morphologically, grade 3 lesions were essentially identical to clear cell EIC, while grade 1 and 2 lesions were designated clear cell EmGD (Figure 2). We tentatively considered these lesions the putative precancerous lesions for ECCC and proceeded to characterize their phenotype. At least one such putative precancer could be identified in 27 of the 30 cases evaluated, with an average of 2.5 foci per case [87]. Immunohistochemical p53 scores (0–9 scale) for these precancers were on average (4.5) lesser than the adjacent carcinomas (6.2) but significantly greater than the benign endometria (0). Similarly, the average proliferative index for the clear cell precancers (45%) was intermediate between the carcinomas (63%) and benign endometria (15%). The lesions also showed notably reduced expression of ER and PR as compared with the adjacent benign endometria [87]. This composite of morphologic and immunophenotypic features resulted in lesions that were notably distinct from the background benign endometrium. In the control group of 38 benign uteri and 30 uteri with endometrioid cancers, no single immunohistochemically-confirmed clear cell precancer was identified [87]. Given their high rate of association with carcinomas having a clear cell component, their lack thereof in the control group of endometrioid cancers, their frequent occurrence in otherwise benign endometria and their aforementioned phenotype, we hypothesized that these lesions represent the precursor lesions of ECCC [87]. It is acknowledged, however, that significantly more research is required to define the full clinicopathologic spectrum of these distinctive lesions and to establish their precancerous nature according to NCI criteria [33].

## Summary

Endometrial carcinomas are remarkably diverse in their biologic behavior. Perhaps more so than other organ systems, the histotype designation alone (endometrioid, serous, clear cell etc) provides a substantial amount of prognostic information. The present nomenclature for putative precursor lesions of endometrial cancers describes an incoherent amalgam that encompasses a lesion with very little malignancy risk (simple hyperplasia without atypia), and on the other end of the "spectrum", serous EIC, a lesion with inherent potential malignancy. Upon superficial inspection, this seems to rightfully mirror the biologic diversity of the invasive cancers for which they probably represent progenitors. However, a critical appraisal quickly reveals that at least a subset of the variability in the malignant potential of these precursors is attributable to definitional problems. Most importantly, as we have previously noted, we believe that the terms "EIC" or "Serous EIC" should be discarded as a means to describe an intraepithelial precancer. These lesions can show extrauterine spread and should thus be considered small uterine serous cancers. None of the other putative endometrial precancers have been reported to show extrauterine spread when present in isolation. For ESCs, we have presented morphologic, immunophenotypic, molecular and follow-up data that strongly suggests that EmGD represents their most probable precancerous lesion. EmGD fulfils most of the NCI requirements for a precancerous lesion [33]. For endometrial clear cell carcinomas, we similarly presented morphologic and immunophenotypic data that suggests that EmGD of the clear cell type represents their precancerous lesion, although there is insufficient data at present time to conclusively establish their precancerous nature by NCI standards.

Much is unknown about the nature, morphologic spectrum and clinical significance of endometrial precancers. However, their eventual characterization will likely start from their morphologic recognition. As such, the lesions described herein are worthy of segregation by pathologists and further study.

## Competing interests

The author(s) declare that they have no competing interests.

## Authors' contributions

OF and WZ co-wrote the manuscript
